# The mediating role of depression in the association between social capital and school absenteeism among students: a multilevel mediation analysis in Japanese elementary and junior high schools

**DOI:** 10.1186/s13034-025-00953-x

**Published:** 2025-08-25

**Authors:** Hiroyuki Mori, Michio Takahashi, Masaki Adachi, Tomoko Nishimura, Hiroki Shinkawa, Makoto Osada, Minami Adachi, Kazuhiko Nakamura

**Affiliations:** 1 Faculty of Humanities, Saitama Gakuen University, 1510 Kizoro, Kawaguchi-shi, Saitama-ken 333-0831 Japan; 2 Research Department, Institute of Child Developmental Science Research, Hamamatsu, Shizuoka Japan; 3 Smart-Aging Research Center, Tohoku University, Sendai, Miyagi Japan; 4 Department of Neuropsychiatry, Graduate School of Medicine, Hirosaki University, Hirosaki, Aomori Japan; 5 Department of Psychology, Meiji Gakuin University, Minato-Ku, Tokyo, Japan; 6 Research Centre for Child Mental Development, Hamamatsu University School of Medicine, Hamamatsu, Shizuoka Japan; 7 United Graduate School of Child Development, The University of Osaka, Kanazawa University, Hamamatsu University School of Medicine, Chiba University and University of Fukui, Suita, Osaka Japan; 8 Faculty of Education, Hirosaki University, Hirosaki, Aomori Japan

**Keywords:** School absenteeism, Social capital, Depression, Multilevel mediation analysis, School climate

## Abstract

**Background:**

School absenteeism is a significant issue affecting children and adolescents worldwide. This study aimed to examine the mediating role of depression in the association between social capital and school absenteeism among students in Japanese elementary and junior high schools.

**Methods:**

A cross-sectional study was conducted using data from 7765 students (aged 9–15 years, 49.7% female) in Hirosaki, Japan. Social capital was measured using the Japanese version of the Social Capital Questionnaire for Adolescent Students (SCQ-AS), depression was assessed via the Patient Health Questionnaire for Adolescents (PHQ-A), and school absence was reported by parents/guardians. Multilevel mediation analysis was employed to examine the associations at both the student and school levels.

**Results:**

At the student level, depression fully mediated the relationship between social capital (school social capital, perceived safety, and neighborhood social capital) and school absence. At the school level, perceived safety had a direct effect on school absence (β = − 0.70, *p* < 0.01), whereas school social capital was negatively associated with depression (β = − 0.57, *p* < 0.001). However, no significant indirect effects were observed at the school level.

**Conclusion:**

Depression mediates the association between social capital and school absence at the student level, whereras school-level perceived safety is directly associated with school absence. These findings suggest a multitiered approach to addressing school absenteeism, focusing on enhancing individual social capital and mental health support, as well as improving school-wide safety perceptions.

## Introduction

School absenteeism is one of the most serious problems for children and adolescents worldwide [1]. In Japan, the number of school absentee students reached a record high in 2022 [2], and Ministry of Education, Culture, Sports, Science and Technology has announced a plan to cope with school absenteeism by emphasizing the importance of school climate, called the"the Comfortable, Customized and Optimized Locations of learning (COCOLO) Plan” [3].

School absenteeism can be a risk factor for school dropout and cause serious problems in mental health and social adjustment in youth and adulthood [4–8]. Many studies have found risk factors related to school absenteeism, such as internalizing and externalizing problems [9], family factors [10], neighborhood or community factors [11], and school factors [9]. There are two perspectives to interpreting school absenteeism: analytic and systemic [12]. The analytic perspective focuses on the experiences and environments that individuals face, such as relationships and communication (microsystem and mesosystem), while the systemic perspective focuses on social structures and culture, such as school climate and educational policy (exosystem and macrosystem [13]). Thus, school and community factors associated with absenteeism can be understood from both perspectives [14]. Furthermore, those factors severely impact vulnerable students, such as students who have psychological vulnerabilities and students from families with low socioeconomic status [15, 16].

Although school and community factors are associated with school absenteeism and the associations are considered robust, it is unclear how these factors contribute to and play a role in school absenteeism. Previous studies indicate that the school environment predict depression and anxiety later in life [17, 18], and then these internalizing problems are risk factors for school absenteeism [9]. Hence, some studies have explored the mechanisms by which the school environment influences student health and, eventually, absenteeism [14]. As the school environment can shape the attitudes and behavior of students and affect their physical and mental health [19, 20], a negative school environment may cause mental health problems such as depression and a lack of energy among students, resulting in school absenteeism. However, no study has identified the role of depression in the mechanism by which the school environment contributes to the development of school absenteeism. Fornander and Kearney [21] found that one particular depression item (nothing much fun anymore) helped demarcate the severity levels of school absenteeism to the greatest extent, suggesting that depression is an early warning sign for youth at risk of severe school absenteeism.

Thus, this study aimed to clarify the mechanisms by which the school environment can contribute to school absenteeism and the role of depression in these mechanisms. A multidimensional, multi-tiered support framework has been proposed for school absenteeism, including preventive interventions such as enhancing school climate and safety, and clinical approaches to mental health problems, but empirical research has not been conducted [22]. Given that school environment integrates both analytic and systemic perspectives, social capital is particularly appropriate for this study. Social capital—which encompasses both individual-level relationships and group-level perceptions of safety and trust, including aspects such as school climate and connectedness– reflects the multidimensional and multi-tiered nature of the school environment [23]. Additionally, social capital is significantly associated with mental health among children and adolescents at the individual and school levels [24]. Identifying the direct effects of the school environment on school absenteeism, or its indirect effects through depression, could provide useful insights for the appropriate implementation of interventions to prevent or mitigate school absenteeism. Based on previous research [14], this study hypothesizes that depression mediates the association between the school environment and school absenteeism. Considering that absenteeism is associated with individual, school, and community factors [25], this study used a multilevel mediation analysis to examine this hypothesis from the analytic and systemic perspectives. To our knowledge, this is the first study to apply multilevel mediation model to a large, population-based sample, enabling the simultaneous estimation of how school environment factors influence absenteeism through depression at both the student and school levels. The findings of this study can contribute to the understanding of the mechanisms and approaches to absenteeism from the analytical and systemic perspectives. The insights may aid the development of policy-level programs targeting school climate and safety, and clinical practices aimed at supporting students experiencing psychological challenges, with the goal of preventing or reducing school absenteeism [26].

## Methods

### Participants and procedures

Data were obtained from the Assessment from Preschool to Puberty—Longitudinal Epidemiological (APPLE) study conducted in Hirosaki, Japan [27]. The study focused on students from the fourth to ninth grades (ages 9–15 years). Parents and guardians were informed of the aims and content of the study and given the opportunity to decline participation before data collection. Students were informed of the study and were explicitly told that they could choose not to participate without any disadvantages. This explanation was provided by their teachers prior to the distribution of the questionnaires. Data were collected during school hours in 2019. The final sample consisted of 7,765 students from 52 schools (35 elementary schools and 17 junior high schools), including 3,769 elementary school students (1,913 male, 1,856 female) and 3,996 junior high school students (1,996 male, 2,000 female). The study was approved by the Committee of Medical Ethics of Hirosaki University Graduate School of Medicine (IRB# 2015–055).

### Measures

#### Outcome variable

School absence was measured using a single item reporting the number of days the student was absent from school during the first semester (April to July). Parents or guardians reported this information on a 6-point scale (0 = nothing, 1 = 1 day, 2 = 2 or 3 days, 3 = 4 to 6 days, 4 = 7 to 9 days, 5 = 10 days or more). Since objective records of student absences from schools were not available, we collected absence data from parent or guardian reports, consistent with previous research (e.g [28]).

#### Independent variables

Social capital was measured using the Japanese version of the Social Capital Questionnaire for Adolescent Students (SCQ-AS [29], Japanese version [30]). The scale consists of 12 items rated on a 3-point scale (1 = “disagree”, 2 = “I do not know, have no opinion”, 3 = “agree”). The total score ranges from 12 to 36, with a higher score indicating greater social capital. The Japanese version has three subscales: “school trust and social cohesion” (eight items; school social capital), “perceived safety in the school and neighborhood” (two items; perceived safety), and “neighborhood trust and social cohesion” (two items; neighborhood social capital) [30].

#### Mediator

Depression was measured using the Japanese version of the Patient Health Questionnaire for Adolescents (PHQ-A [31], Japanese version [32]). The PHQ-A consists of nine items rated on a 4-point scale (0 = “not at all” to 3 = “nearly every day”). Total scores range from 0 to 27, with a higher score indicating stronger depressive symptoms.

#### Covariates

Student-level covariates included grade (4th to 9th) and sex (0 = male, 1 = female). These were included as potentially confounding variables as research suggests that junior high school students and females tend to experience more severe depression [33].

### Statistical analysis

#### Missing data

The full information maximum likelihood (FIML) method was used to control for missing data [34], which can generate unbiased and effective parameter estimates based on complete data information.

#### Multilevel mediation modeling

**Fig. 1:**
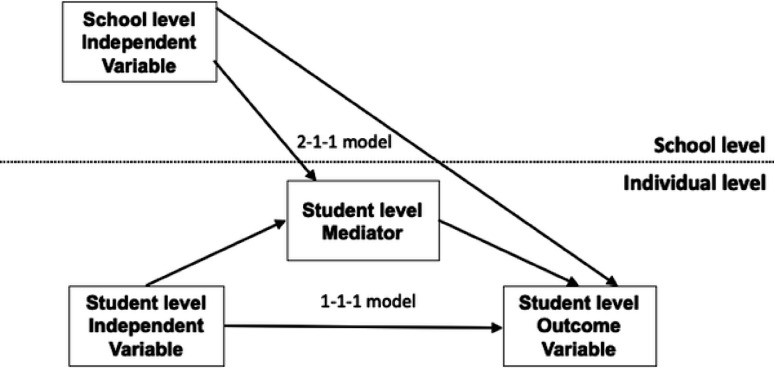
Conceptual model of the multilevel mediation analysis

Multilevel mediation modeling was used to simultaneously examine the direct and indirect effect of social capital on school absence through depression at the student and school level. In our model, the independent variable was at the student and school level, and the outcome variable and the mediator were at the student level, and – a so-called 1-1-1 and 2-1-1 design (Fig. 1). School-level aggregated scores were calculated by averaging student-reported scores on each subscale of the SCQ-AS. These were used as school means, representing students’ shared perceptions of school-wide social capital. Group-mean centering by school was utilized for student-level predictors, and grand-mean centering was utilized for school-level predictors, as recommended for research questions comparing the effects of student-level predictors and their corresponding higher-level predictors [35]. These strategies help distinguish within-group (student-level) and between-group (school-level) effects and are essential for accurate interpretation of multilevel mediation paths by reducing potential confounding due to group-level variance [35].

#### Preliminary analysis

First, the intraclass correlation coefficients (ICCs) of the main variables were computed using an unconditional model to determine the variance attributable to student and school level differences. Subsequently, the design effects (DEFF) for each variable were analyzed to assess the need for multilevel modeling [36]. A DEFF greater than 1.1 indicates that multilevel modeling is necessary when estimating the effects of group-level predictors.

#### Main analyses

In Step 1, the independent variables (school social capital, perceived safety, and neighborhood social capital at both student and school levels) were inputted into the model with school absence as the outcome variable. In Step 2, the same independent variables as Step 1 were used, with depression as the dependent variable. Finally, in Step 3, each social capital component and depression were entered into the model, as independent variables and a mediator, respectively, with school absence as the outcome variable.

Model fit was evaluated using the Deviance Information Criterion (DIC), with ΔDIC > 7 considered to indicate better fit [37]. All analyses were performed using Mplus version 8.10 [38]. The significance threshold was set at *p* < 0.05.

## Results

### Preliminary analyses

The descriptive statistics of the variables are reported in Table 1.

**Table 1:** Descriptive statistics of independent variables, mediator and covariates

Student characteristics	Elementary school			Junior high school		
	*n* = 3769			*n* = 3996		
	M (SD)	Min	Max	M (SD)	Min	Max
Grade	8.01 (0.82)	4	6	8.00(0.82)	7	9
Sex (Female)	49.24%	0	1	50.05%	0	1
PHQ-A	4.13 (4.70)	0	27	4.63 (4.82)	0	27
SCQ-AS total score	31.03 (4.11)	13	36	31.09 (4.08)	14	36
School social capital	20.71 (3.01)	8	24	20.92 (2.96)	8	24
Perceived safety	5.35 (0.97)	2	6	5.32 (1.02)	2	6
Neighborhood social capital	4.97 (1.10)	2	6	4.84 (1.13)	2	6

School characteristics	*n* = 35			*n* = 17		
	M (SD)	Min	Max	M (SD)	Min	Max
SCQ-AS total score	31.44 (1.25)	29.19	34.55	31.28 (0.70)	30.38	32.55
School social capital	20.97 (0.89)	19.21	23.41	21.01 (0.45)	20.34	21.72
Perceived safety	5.40 (0.19)	5.08	5.82	5.37 (0.14)	5.20	5.67
Neighborhood social capital	5.05 (0.26)	4.55	5.67	4.91 (0.25)	4.50	5.30

Before conducting the main analyses, the ICCs and DEFF for the main variables were computed. The ICCs indicated that 0.01 of the variance in the outcome variables was attributable to school-level differences. The DEFF values were greater than 1.1 for 2.21, confirming the need for multilevel modeling.

### Multilevel mediation analysis

The results of multilevel mediation analysis are presented in Table 2. The model fit improved from Step 1 to Step 3, as indicated by the decrease in the Deviance Information Criterion (DIC) from 60729.75 to 57947.65. The final model (Step 3) explained 2% of the variance in school absence at the individual level and 69% at the school level, suggesting that much of absenteeism may be shaped by school climate factors.

**Table 2:** Multilevel mediation analysis: association between social capital and school absence through depression

	Step 0	Step 1	Step 2	Step 3	
	School absence	School absence	Depression	Depression	School absence
		β (SE)	β (SE)	β (SE)	β (SE)
*Student-level*					
Sex		–0.03 (0.01)**	0.03 (0.01)*		–0.04 (0.01)**
Grade		–0.04 (0.01)***	0.02 (0.02)		–0.05 (0.02)**
School social capital		–0.05 (0.01)***	–0.48 (0.01)***	–0.48 (0.01)***	–0.00 (0.02)
Perceived safety		–0.02 (0.01)	–0.13 (0.01)***	–0.13 (0.01)***	–0.01 (0.01)
Neighborhood social capital		–0.02 (0.01)	–0.04 (0.01)***	–0.04 (0.01)***	–0.02 (0.01)
*School-level*					
School social capital		0.24 (0.22)	–0.53 (0.18)***	–0.57(0.20)**	0.08 (0.28)
Perceived safety		–0.68 (0.17)**	–0.19 (0.22)	–0.17 (0.21)	–0.70 (0.17)**
Neighborhood social capital		0.19 (0.23)	–0.35 (0.20)	–0.31 (0.21)	0.06 (0.27)
*Student-level mediator*					
Depression in the 1-1-1 model					0.11 (0.01)***
Depression in the 2-1-1 model					–0.31 (0.30)
*Model Information criteria*					
R^2^ at individual-level		0.01***	0.31***	0.30***	0.02***
R^2^ at school-level		0.65***	0.54***	0.55***	0.69***
Deviance (DIC)	16867.75	60729.75	58068.16	57947.65	

^*^p < 0.05, ^**^p < 0.01, ^***^p < 0.001

#### Direct effects on school absence (step 1)

At the student level, school social capital displayed a significant negative association with school absence (β = −0.05, *p* < 0.001), while perceived safety and neighborhood social capital showed no significant association. At the school level, only perceived safety displayed a significant association with school absence (β = −0.68, *p* < 0.01).

#### Effects of social capital on depression (step 2)

All three dimensions of social capital at the student level were significantly negatively associated with depression: school social capital (β = −0.48, *p* < 0.001), perceived safety (β = −0.13, *p* < 0.001), and neighborhood social capital (β = −0.04, *p* < 0.001). At the school level, only school social capital showed a significant negative association with depression (β = −0.53, *p* < 0.001).

#### Mediating effects of depression on the association between social capital and school absence (step 3)

In the final step of the multilevel mediation analysis, depression displayed a significant positive association with school absence in the 1-1-1 model (β = 0.11, *p* < 0.001). The direct effects of school social capital on school absence became non-significant at the student level when depression was included in the model, indicating that depression fully mediated the association between social capital and school absence. The indirect effect of school social capital (b = − 0.02, *p* < 0.001), perceived safety (b = − 0.01, *p* < 0.001), and neighborhood social capital (b = − 0.00, *p* < 0.001) on school absence through depression were observed at the student level.

In the 2-1-1 model, school social capital at the school level was negatively associated with depression (β = −0.57, *p* < 0.01) and the direct effect of perceived safety on school absence remained significant (β = −0.70, *p* < 0.01), but depression was not significantly associated with school absence. At the school level, no significant indirect effects of social capital on school absence through depression were observed. Moreover, the multi-level mediation analysis showed that at the school level, the rate of explanation for school absence was adequate (69%), whereas it was low (2%) at the student level.

These findings suggest that social capital, particularly at the student level, may influence school absenteeism, primarily through its effect on depression. The role of social capital at the school level may be more complex, as its direct effects on depression and absenteeism vary across social capital subcomponents, but do not display any significant indirect effects on absenteeism via depression.

## Discussion

This study aimed to clarify the mechanisms by which school social capital contributes to school absenteeism and the mediating role of depression in these mechanisms. The findings indicate significant indirect effects of all three dimensions of social capital on school absence through depression at the student level. At the school level, while school social capital was negatively associated with depression, no significant indirect effects on school absence were observed. However, perceived safety at the school level had a direct effect on school absence. These findings suggest that the mechanisms by which social capital and school absenteeism are associated may differ at the student and school levels in Japanese elementary and junior high schools.

### Multilevel mediation analysis for 1-1-1 model

The results demonstrate that social capital at the student level is not directly associated with school absence, but primarily influences school absence through depression. This indicates that a positive school environment influences child development and educational outcomes [39]. It also provides empirical evidence for this association and extends previous research by Fornander and Kearney [21], which suggests that depression might be an early warning sign for youth at risk of severe school absenteeism. Moreover, the findings indicate that at the student level, school social capital is more strongly associated with depression than perceived safety and neighborhood social capital. This is consistent with a previous study involving Japanese elementary and junior high school students [24].

### Multilevel mediation analysis for 2-1-1 model

Notably, the role of school-level social capital appears to be more complex. While school social capital at the school level was negatively associated with depression, no significant indirect effects on school absence through depression were observed at the school level. The greater association of school social capital with depression, similar to the student level, underscores the crucial role of the school environment in affecting students’ developmental and mental health [17].

The significant direct effect of perceived safety on school absence at the school level, even after controlling for depression, highlights the importance of school and community safety in reducing absenteeism. The association between school level perceived safety and school absence observed in this study aligns with Kearney et al. [1] emphasis on the role of perceptions of school and community safety in the systemic perspective of school absenteeism. It also supports the suggestion of a previous study that safe and supportive schools enhance students’ sense of belonging, resulting in higher school attendance [40].

This study is among the first to apply multilevel mediation analysis to a large population-based sample of students in Japan to examine both student- and school-level mechanisms underlying absenteeism. The differences in the mediating role of depression on the association between social capital and school absence suggest that the school environment’s impact on absenteeism may function through different mechanisms at the school level compared to the student level. Interestingly, the rate of explanation for absenteeism was much higher at the school level than at the individual level. These results are consistent with previous studies showing that school factors play significant roles in school absenteeism [9, 41]. The findings also suggest that, regardless of the severity of depression, a higher average perception of school safety has a positive effect on encouraging students to attend school and reducing the number of days absent. In addition, school climate, a presentation of school social capital, is generated by individual-level perceptions but has a collective effect on students that exceeds individual experience [19], suggesting that the influence of school climate on students’ mental health and attendance is a complex process. The findings of this study support the multidimensional, multi-tiered systems of support framework proposed for absenteeism [22]. From an analytical perspective, the results underscore the importance of focusing on individual experiences and environments, particularly in terms of relationships and communication within the school [12, 13]. Interventions aimed at enhancing students’ perceptions of school social capital and safety could reduce depressive symptoms and, consequently, absenteeism. From a systemic perspective, the findings highlight the need to address school climate and safety at a broader level [12]. The direct effect of school-level perceived safety on absenteeism suggests that school-wide approaches to improve safety could significantly reduce absenteeism, regardless of their effects on individual students’ mental health. Whole-school initiatives such as cooperative learning and cross-grade activities have been shown to enhance students’ sense of belonging and trust [42]. In addition, school-wide PBIS interventions have improved perceptions of safety and behavioral norms [43], which may help reduce depression and absenteeism.

### Strength and limitations

This study has several strengths as the first study clarifies the mediating role of depression in the association between social capital and school absence. Additionally, the multilevel mediation analysis of the 1-1-1 model and 2-1-1 model allowed the simultaneous estimations of direct and indirect effects of social capital on school absence at both the student and school level.

However, this study has several limitations. First, its cross-sectional nature limits the ability to establish causal relationships. Longitudinal studies are needed to confirm the temporal sequence of social capital, depression, and school absence. Second, the measure of school absence was based on a single item reported by parents or guardians, which may not capture the full complexity of school attendance patterns. Data from students who did not attend school enough may not have been obtained as the questionnaires were distributed in the classroom. Third, this study did not include family relationships and personal characteristics (e.g., resilience), which are significantly linked to mental health outcomes [44]. Furthermore, objective assessments by parents and teachers, which could have provided a more comprehensive view of students’ mental health and social capital, were not included [45]. Future research should consider including these factors—such as resilience, parental support, and teacher-reported student engagement— to provide a more holistic understanding of the relationship between social capital and mental health among adolescents. Finally, the multi-level mediation analysis showed that at the school level, the rate of explanation for absenteeism was adequate (69%), whereas at the student level, the rate of explanation for absenteeism was low (2%). This suggests that school-level factors may play a more significant role in school absenteeism. However, at the student level, better explanatory models should be considered that include the factors mentioned above. A previous study reported that school factors could reduce school absenteeism by preventing bullying and social exclusion by peers [41], suggesting that some confounding factors can exist in the associations between school factors and school absenteeism. Further research is needed to explore the associations between school factors and school absenteeism, including other mediators at the student level.

## Conclusion

This study revealed that depression mediates the association between social capital and school absence at the student level, while school-level perceived safety of social capital is directly associated with school absence. These findings suggest the need for a multi-tiered approach to address absenteeism that enhances individual social capital and mental health support, while simultaneously improving school-wide safety perceptions. This comprehensive strategy can aid the development of effective interventions—such as school climate improvement programs or school-based mental health screening—leading to improved educational outcomes and enhanced student well-being.

## Data Availability

No datasets were generated or analysed during the current study.

## Abbreviations

COCOLO Plan: Comfortable, Customized and Optimized Locations of learning Plan
APPLE study: Assessment from Preschool to Puberty - Longitudinal Epidemiological study
SCQ-AS: Social Capital Questionnaire for Adolescent Students
PHQ-A: Patient Health Questionnaire for Adolescents
FIML: Full information maximum likelihood
ICCs: Intraclass correlation coefficients
DEFF: Design effects
DIC: Deviance Information Criterion
